# Supplementary material for: Bidirectional Associations between Daytime Napping Duration and Metabolic Syndrome: A Nationally Representative Cohort Study
Source: Nutrients. 2022 Dec 12;14(24):5292. doi: 10.3390/nu14245292 (PMC9785410; doi:10.3390/nu14245292)
Supplement: Supplementary file 1 [file nutrients-14-05292-s001.zip › nutrients-2025494-supplementary.pdf]

Supplementary Materials

Supplemental Methods. The definition of MetS based on the criteria of International Diabetes Federation (IDF)

Figure S1. Study flowchart of participant selection in three subcohorts

Figure S2. The time line of the study

Figure S3. Longitudinal dose-response relationship between baseline daytime napping duration and the remission of MetS

Table S1. Baseline characteristics of 5041 participants according to daytime napping duration in the subcohort 1

Table S2. Baseline characteristics of 2898 participants according to daytime napping duration in the subcohort 2

Table S3. Baseline characteristics of 11390 participants according to metabolic syndrome status in the subcohort 3

Table S4. Longitudinal associations of baseline daytime napping duration with the occurrence of MetS components

Table S5. Longitudinal associations of baseline daytime napping duration with the remission of MetS components

Table S6. Longitudinal associations of baseline daytime napping duration with the occurrence and remission of MetS after further adjusting for physical activity and multiple drug therapy

Table S7. Longitudinal associations of baseline daytime napping duration with the occurrence and remission of MetS according to inverse probability of treatment weighting

Table S8. Longitudinal associations of baseline daytime napping duration with the occurrence and remission of MetS after changing the diagnostic criteria of MetS

Table S9. Association of daytime napping duration with MetS severity score according to ordinal logistic regression

Table S10. Longitudinal associations of baseline daytime napping duration with the occurrence and remission of MetS in subgroups by depression, age and gender

Table S11. Longitudinal associations of baseline metabolic syndrome status and its components with follow-up daytime napping duration after further adjusting for physical activity and multiple drug therapy

Table S12. Longitudinal associations of baseline metabolic syndrome status and its components with follow-up daytime napping duration after using the secondary outcomes

Table S13. Longitudinal associations of baseline metabolic syndrome status and its components with follow-up daytime napping duration in subgroup analyses

Table S14. Cross-lagged regression coefficient of daytime napping duration with MetS severity score

Supplemental Methods. The definition of MetS based on the criteria of International Diabetes Federation (IDF)

According this definition[33], for a person to be defined as having the MetS, they must have central obesity (waist circumference ≥90 cm for men and ≥85 cm for women) plus any two or more of four additional factors. These four factors are: (1) systolic blood pressure ≥130 mmHg or diastolic blood pressure ≥85 mmHg or self-reported hypertension or using antihypertensive drugs; (2) fasting plasma glucose (FPG) ≥5.6 mmol/L or self-reported diabetes or the use of diabetes medication.; (3) reduced plasma HDL-C (<1.0 mmol/L for men and <1.3 mmol/L for women) or specific treatment for this lipid abnormality; and (4) elevated plasma TG (≥1.7 mmol/L) or specific treatment for this lipid abnormality.

Reference

33. Alberti, K.G.; Zimmet, P.; Shaw, J. Metabolic syndrome--a new world-wide definition. A Consensus Statement from the International Diabetes Federation. Diabet Med. **2006**, 23, 469-480. https://doi.org/10.1111/j.1464-5491.2006.01858.x.

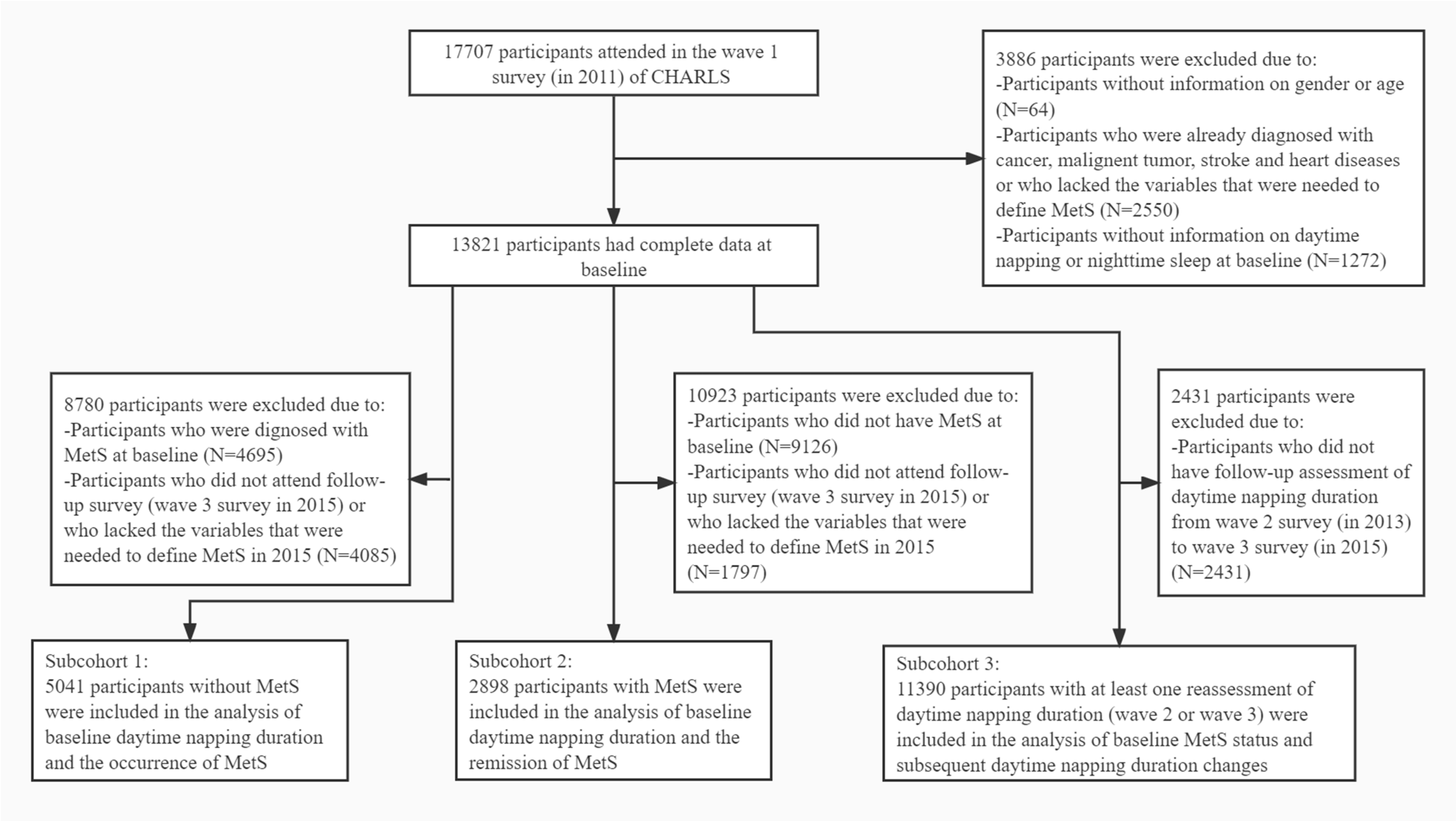

Figure S1. Study flowchart of participant selection in three subcohorts.

Abbreviations: MetS, metabolic syndrome; CHARLS, China Health and Retirement Longitudinal Study.

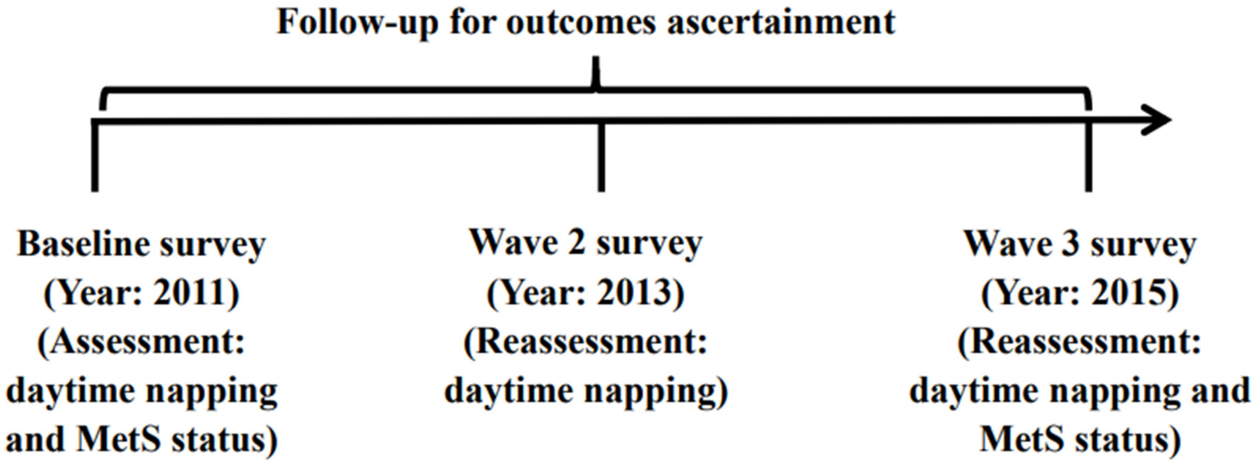

Figure S2. The time line of the study
Abbreviations: MetS, metabolic syndrome

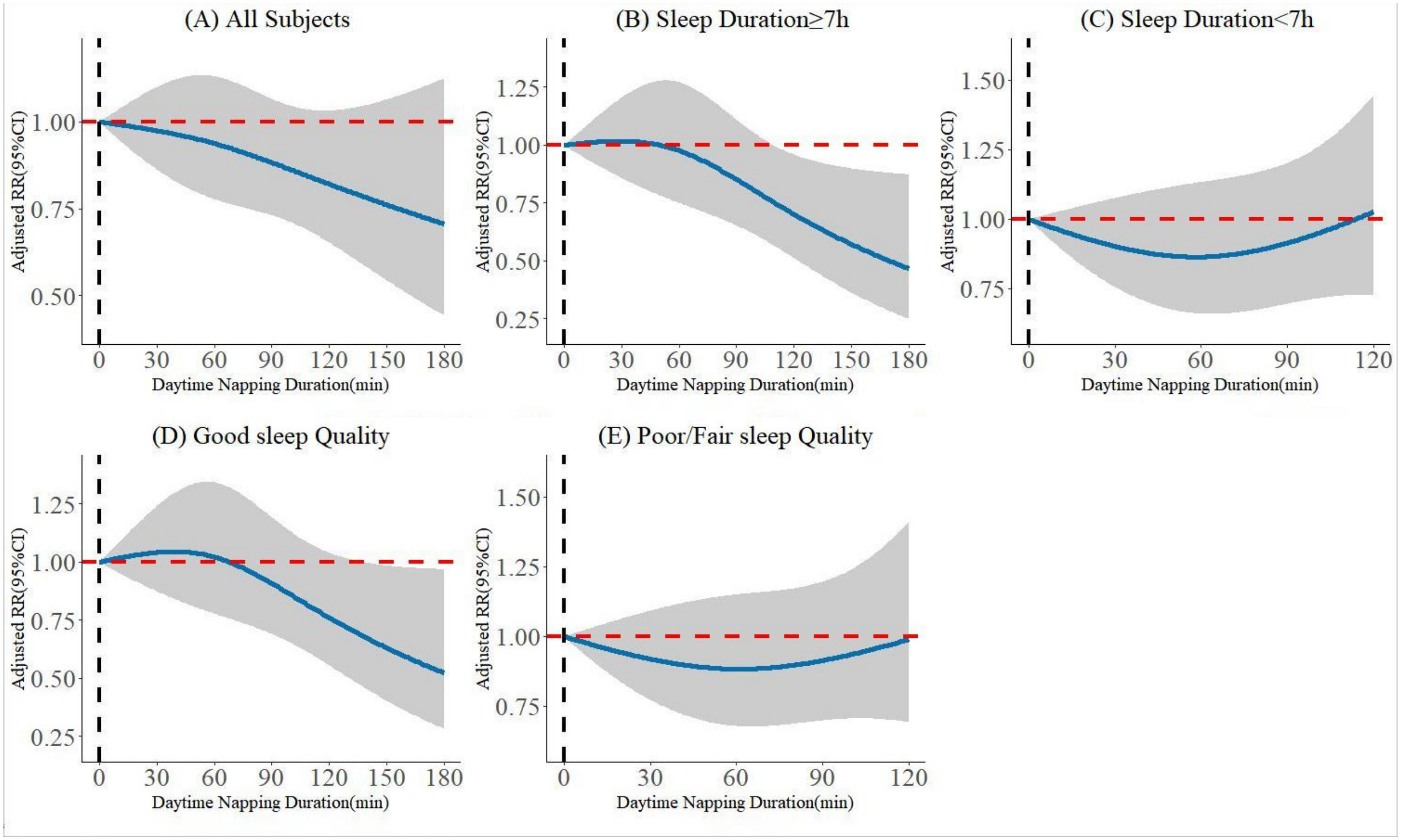

Figure S3. Longitudinal dose-response relationship between baseline daytime napping duration and the remission of MetS

The curve was estimated by restricted cubic spline function with four knots. Solid lines indicate aRR. The reference was set to 0 min. The shadow represents

95% confidence intervals.

Table S1. Baseline characteristics of 5041 participants according to daytime napping duration in the subcohort 1

Characteristics	Daytime napping duration				*P* value
	0 minute/day	≤30 minutes/day	30–90 minutes/day	>90 minutes/day	
**No. of participants**	2515	458	1405	663	
**Age (years), mean (SD)**	57.70 (8.89)	56.67 (8.73)	58.32 (9.33)	58.58 (9.69)	0.001
**Male, n (%)**	1185 (47.1)	212 (46.3)	905 (64.4)	409 (61.7)	<0.001
**Married, n (%)**	2152 (85.6)	402 (87.8)	1225 (87.2)	554 (83.6)	0.090
**Elementary school or above, n (%)**	1305 (51.9)	255 (55.7)	840 (59.8)	408 (61.5)	<0.001
**Rural residence, n (%)**	2207 (87.8)	361 (78.8)	1202 (85.6)	567 (85.5)	<0.001
**Smoking status, n (%)**					<0.001
Current smoker	821 (32.6)	132 (28.8)	589 (41.9)	290 (43.7)	
Former smoker	168 (6.7)	26 (5.7)	138 (9.8)	58 (8.7)	
Non-smoker	1526 (60.7)	300 (65.5)	678 (48.3)	315 (47.5)	
**Drinking status, n (%)**					<0.001
More than once a month	625 (24.9)	127 (27.7)	503 (35.8)	235 (35.4)	
Drink but less than once a month	195 (7.8)	40 (8.7)	126 (9.0)	56 (8.4)	
Never	1695 (67.4)	291 (63.5)	776 (55.2)	372 (56.1)	
**Physical activity, n (%)**					0.010
None	252 (10.0)	48 (10.5)	133 (9.5)	88 (13.3)	
Mild	462 (18.4)	99 (21.6)	288 (20.5)	152 (22.9)	
Moderate	762 (30.3)	141 (30.8)	420 (29.9)	197 (29.7)	
Vigorous	1039 (41.3)	170 (37.1)	564 (40.1)	226 (34.1)	
**Depressive symptoms, n (%)**	987 (39.2)	162 (35.4)	477 (34.0)	237 (35.7)	0.007
**BMI (kg/m^2), mean (SD)**	22.89 (4.30)	23.05 (4.28)	23.26 (4.31)	23.61 (4.52)	0.001
**Uric acid (mg/dL), mean (SD)**	4.14 (1.15)	4.14 (1.17)	4.48 (1.31)	4.54 (1.35)	<0.001

**HsCRP (mg/L), median [IQR]**	0.96 [0.51, 2.39]	0.81 [0.45, 1.82]	0.81 [0.47, 1.75]	0.74 [0.47, 1.88]	<0.001
**LDL-C (mg/dL), mean (SD)**	101.02 (38.48)	98.10 (38.37)	98.63 (42.37)	96.57 (44.35)	0.041
**Antihypertensive agents, n (%)**	180 (7.2)	26 (5.7)	95 (6.8)	48 (7.2)	0.687
**Hypoglycemic agents, n (%)**	21 (0.8)	5 (1.1)	13 (0.9)	6 (0.9)	0.904
**Lipid-lowering agents, n (%)**	10 (0.4)	1 (0.2)	7 (0.5)	3 (0.5)	0.882
**sleeping pills/ anti-depressive treatment, n (%)**	19 (0.8)	1 (0.2)	3 (0.2)	7 (1.1)	0.036
**Good sleep quality, n (%)**	1163 (46.2)	216 (47.2)	759 (54.0)	380 (57.3)	<0.001
**Nighttime sleep duration (h), mean (SD)**	6.29 (1.96)	6.28 (1.73)	6.54 (1.74)	6.63 (1.96)	<0.001
**Outcome variable**					
The incidence of MetS, n (%)	556 (22.1)	105 (22.9)	300 (21.4)	165 (24.9)	0.329

Data are presented as the mean (SD), median [IQR] or number (%), as appropriate. Continuous variables were compared using one-way ANOVA test or Kruskal-Wallis test. Categorical variables were compared using χ^2 test or Fisher's exact test.

Abbreviations: SD, standard deviation; IQR, interquartile range; BMI, body mass index; MetS, metabolic syndrome; LDL-C, low-density lipoprotein cholesterol; hsCRP, high sensitivity C-reactive protein.

Table S2. Baseline characteristics of 2898 participants according to daytime napping duration in the subcohort 2

Characteristics	Daytime napping duration				*P* value
	0 minute/day	≤30 minutes/day	30–90 minutes/day	>90 minutes/day	
**No. of participants**	1273	301	901	423	
**Age (years), mean (SD)**	58.57 (8.87)	58.37 (8.21)	58.69 (8.72)	58.20 (8.98)	0.792
**Male, n (%)**	333 (26.2)	101 (33.6)	378 (42.0)	194 (45.9)	<0.001
**Married, n (%)**	1068 (83.9)	256 (85.0)	765 (84.9)	369 (87.2)	0.428
**Elementary school or above, n (%)**	601 (47.2)	163 (54.2)	491 (54.5)	241 (57.0)	<0.001
**Rural residence, n (%)**	1047 (82.2)	229 (76.1)	711 (78.9)	358 (84.6)	0.007
**Smoking status, n (%)**					<0.001
Current smoker	254 (20.0)	53 (17.6)	223 (24.8)	126 (29.8)	
Former smoker	56 (4.4)	22 (7.3)	85 (9.4)	36 (8.5)	
Non-smoker	963 (75.6)	226 (75.1)	593 (65.8)	261 (61.7)	
**Drinking status, n (%)**					<0.001
More than once a month	223 (17.5)	61 (20.3)	187 (20.8)	107 (25.3)	
Drink but less than once a month	79 (6.2)	25 (8.3)	88 (9.8)	31 (7.3)	
Never	971 (76.3)	215 (71.4)	626 (69.5)	285 (67.4)	
**Physical activity, n (%)**					<0.001
None	219 (17.2)	23 (7.6)	96 (10.7)	50 (11.8)	
Mild	311 (24.4)	87 (28.9)	266 (29.5)	131 (31.0)	
Moderate	401 (31.5)	109 (36.2)	291 (32.3)	135 (31.9)	
Vigorous	342 (26.9)	82 (27.2)	248 (27.5)	107 (25.3)	
**Depressive symptoms, n (%)**	526 (41.3)	109 (36.2)	283 (31.4)	112 (26.5)	<0.001
**BMI (kg/m^2), mean (SD)**	25.73 (3.87)	25.46 (3.67)	25.92 (3.68)	26.20 (3.62)	0.039
**Uric acid (mg/dL), mean (SD)**	4.34 (1.38)	4.48 (1.43)	4.91 (1.66)	4.67 (1.56)	<0.001

**HsCRP (mg/L), median [IQR]**	1.30 [0.67, 2.41]	1.24 [0.68, 2.34]	1.30 [0.67, 2.49]	1.43 [0.74, 2.88]	0.099
**LDL-C (mg/dL), mean (SD)**	127.23 (49.40)	133.44 (49.93)	136.95 (59.23)	133.68 (58.61)	<0.001
**Antihypertensive agents, n (%)**	324 (25.5)	86 (28.6)	292 (32.4)	153 (36.2)	<0.001
**Hypoglycemic agents, n (%)**	81 (6.4)	22 (7.3)	75 (8.3)	27 (6.4)	0.327
**Lipid-lowering agents, n (%)**	111 (8.7)	47 (15.6)	117 (13.0)	43 (10.2)	<0.001
**sleeping pills/ anti-depressive treatment, n (%)**	1 (0.1)	4 (1.3)	4 (0.4)	4 (0.9)	0.005
**Good sleep quality, n (%)**	560 (44.0)	139 (46.2)	474 (52.6)	269 (63.6)	<0.001
**Nighttime sleep duration (h), mean (SD)**	6.23 (1.85)	6.19 (1.74)	6.59 (1.77)	6.99 (1.94)	<0.001
**Outcome variable**					
The reversion of MetS, n (%)	370 (29.1)	90 (29.9)	257 (28.5)	111 (26.2)	0.673

Data are presented as the mean (SD), median [IQR] or number (%), as appropriate. Continuous variables were compared using one-way ANOVA test or Kruskal-Wallis test. Categorical variables were compared using χ^2 test or Fisher's exact test.

Abbreviations: SD, standard deviation; IQR, interquartile range; BMI, body mass index; MetS, metabolic syndrome; LDL-C, low-density lipoprotein cholesterol; hsCRP, high sensitivity C-reactive protein.

Table S3. Baseline characteristics of 11390 participants according to metabolic syndrome status in the subcohort 3

Characteristics	Metabolic syndrome		*P* value
	No	Yes	
**No. of participants**	7454	3936	
**Age (years), mean (SD)**	57.69 (9.35)	58.69 (9.17)	<0.001
**Male, n (%)**	4027 (54.0)	1399 (35.5)	<0.001
**Married, n (%)**	6319 (84.8)	3317 (84.3)	0.499
**Elementary school or above, n (%)**	4199 (56.3)	2055 (52.2)	<0.001
**Rural residence, n (%)**	6282 (84.3)	3080 (78.3)	<0.001
**Smoking status, n (%)**			<0.001
Current smoker	2698 (36.2)	886 (22.5)	
Former smoker	543 (7.3)	295 (7.5)	
Non-smoker	4213 (56.5)	2755 (70.0)	
**Drinking status, n (%)**			<0.001
More than once a month	2186 (29.3)	814 (20.7)	
Drink but less than once a month	606 (8.1)	282 (7.2)	
Never	4662 (62.5)	2840 (72.2)	
**Physical activity, n (%)**			<0.001
None	844 (11.3)	563 (14.3)	
Mild	1450 (19.5)	983 (25.0)	
Moderate	2190 (29.4)	1234 (31.4)	
Vigorous	2970 (39.8)	1156 (29.4)	
**Depressive symptoms, n (%)**	2437 (34.6)	1268 (34.1)	0.579
**BMI (kg/m^2), mean (SD)**	22.70 (4.28)	25.38 (3.78)	<0.001
**Uric acid (mg/dL), mean (SD)**	4.35 (1.20)	4.56 (1.28)	<0.001
**HsCRP (mg/L), median [IQR]**	0.87 [0.49, 1.86]	1.26 [0.66, 2.55]	< 0.001
**LDL-C (mg/dL), mean (SD)**	118.08 (33.77)	118.96 (38.30)	0.204
**Antihypertensive agents, n (%)**	524 (7.0)	1146 (29.1)	<0.001
**Hypoglycemic agents, n (%)**	62 (0.8)	273 (6.9)	<0.001
**Lipid-lowering agents, n (%)**	30 (0.4)	396 (10.1)	<0.001
**sleeping pills/ anti-depressive treatment, n (%)**	43 (0.6)	23 (0.6)	>0.999
**Good sleep quality, n (%)**	3783 (50.8)	1987 (50.5)	0.800
**Nighttime sleep duration (h), mean (SD)**	6.41 (1.88)	6.45 (1.83)	0.274
**Napping duration in 2011 (min/day), mean (SD)**	35.96 (43.87)	40.04 (44.31)	<0.001
**Outcome variables**			
Napping duration in 2013 (min/day), mean (SD)	41.35 (46.38)	44.18 (46.10)	0.003
Napping duration in 2015 (min/day), mean (SD)	41.41 (46.22)	44.25 (45.87)	0.002

Data are presented as the mean (SD), median [IQR] or number (%), as appropriate. Continuous variables were compared using one-way ANOVA test or Kruskal-Wallis test. Categorical variables were compared using χ^2 test or Fisher's exact test.

Abbreviations: SD, standard deviation; IQR, interquartile range; BMI, body mass index; MetS, metabolic syndrome; LDL-C, low-density lipoprotein cholesterol; hsCRP, high sensitivity C-reactive protein.

Table S4. Longitudinal associations of baseline daytime napping duration with the occurrence of MetS components

	N	Case, n (%)	aRR (95% CI)	*P*
**Occurrence of MetS components**				
**Hyperglycaemia**	3650	664 (18.2)		
per-ten minutes increase			1.002 (0.986, 1.017)	0.853
non-nappers [a]	1876	328 (17.5)	1 (ref.)	-
short nappers [a]	333	62 (18.6)	1.035 (0.808, 1.324)	0.787
moderate nappers [a]	964	177 (18.4)	0.980 (0.826, 1.162)	0.815
extended nappers [a]	477	97 (20.3)	1.064 (0.864, 1.311)	0.557
**Hypertriglyceridemia**	4719	1115 (23.6)		
per-ten minutes increase			1.008 (0.997, 1.020)	0.162
non-nappers [a]	2355	537 (22.8)	1 (ref.)	-
short nappers [a]	428	120 (28.0)	1.199 (1.012, 1.420)	**0.036**
moderate nappers [a]	1316	312 (23.7)	1.114 (0.984, 1.260)	0.089
extended nappers [a]	620	146 (23.5)	1.106 (0.941, 1.300)	0.221
**Low HDL-C**	2984	546 (18.3)		
per-ten minutes increase			0.999 (0.982, 1.017)	0.940
non-nappers [a]	1457	277 (19.0)	1 (ref.)	-
short nappers [a]	254	56 (22.0)	1.152 (0.897, 1.480)	0.267
moderate nappers [a]	878	144 (16.4)	0.992 (0.827, 1.190)	0.929
extended nappers [a]	395	69 (17.5)	1.040 (0.825, 1.312)	0.739
**Hypertension**	3527	1022 (29.0)		
per-ten minutes increase			0.989 (0.977, 1.001)	0.066
non-nappers [a]	1740	507 (29.1)	1 (ref.)	-
short nappers [a]	331	94 (28.4)	0.966 (0.802, 1.164)	0.716
moderate nappers [a]	1010	303 (30.0)	0.958 (0.849, 1.082)	0.490
extended nappers [a]	446	118 (26.5)	0.848 (0.715, 1.005)	0.057
**Central obesity**	3897	1071 (27.5)		
per-ten minutes increase			1.014 (1.003, 1.025)	**0.010**
non-nappers [a]	1917	529 (27.6)	1 (ref.)	-
short nappers [a]	360	115 (31.9)	1.042 (0.893, 1.216)	0.603
moderate nappers [a]	1110	283 (25.5)	1.122 (0.995, 1.265)	0.061
extended nappers [a]	510	144 (28.2)	1.204 (1.038, 1.397)	**0.014**

Abbreviations: CI, confidence interval; aRR, adjusted relative risk; MetS, metabolic syndrome; LDL-C, low-density lipoprotein cholesterol; hsCRP, high sensitivity C-reactive protein; HDL-C, high-density lipoprotein cholesterol.

[a] Daytime napping duration of non-nappers:0 minutes/day; Daytime napping duration of short nappers:>0 minutes/day to ≤30 minutes/day; Daytime napping duration of moderate nappers:>30 minutes/day to ≤90 minutes/day; Daytime napping duration of extended nappers:>90minutes/day.

Adjusted for: age, gender, living residence, marital status, educational level, smoking status, drinking status, depressive symptoms, nighttime sleep quality, nighttime sleep duration, serum uric acid, LDL-C , hsCRP and other MetS components.

Table S5. Longitudinal associations of baseline daytime napping duration with the remission of MetS components

	N	Case, n (%)	aRR (95% CI)	*P*
**Remission of MetS components**				
**Hyperglycaemia**	1890	872 (46.1)		
per-ten minutes increase			0.999 (0.988, 1.010)	0.799
non-nappers [a]	827	387 (46.8)	1 (ref.)	-
short nappers [a]	199	98 (49.2)	1.069 (0.912, 1.251)	0.411
moderate nappers [a]	578	253 (43.8)	0.966 (0.859, 1.088)	0.570
extended nappers [a]	286	134 (46.9)	1.035 (0.896, 1.197)	0.639
**Hypertriglyceridemia**	1713	399 (23.3)		
per-ten minutes increase			0.996 (0.977, 1.016)	0.699
non-nappers [a]	732	177 (24.2)	1 (ref.)	-
short nappers [a]	191	42 (22.0)	0.945 (0.702, 1.273)	0.711
moderate nappers [a]	528	119 (22.5)	0.963 (0.786, 1.180)	0.716
extended nappers [a]	262	61 (23.3)	0.989 (0.765, 1.278)	0.932
**Low HDL-C**	2413	749 (31.0)		
per-ten minutes increase			0.994 (0.980, 1.008)	0.379
non-nappers [a]	1043	319 (30.6)	1 (ref.)	-
short nappers [a]	247	78 (31.6)	0.999 (0.813, 1.227)	0.991
moderate nappers [a]	765	246 (32.2)	0.960 (0.834, 1.105)	0.568
extended nappers [a]	358	106 (29.6)	0.884 (0.734, 1.064)	0.192
**Hypertension**	2236	322 (14.4)		
per-ten minutes increase			0.968 (0.945, 0.992)	**0.009**
non-nappers [a]	980	161 (16.4)	1 (ref.)	-
short nappers [a]	226	30 (13.3)	0.833 (0.581, 1.195)	0.322
moderate nappers [a]	696	98 (14.1)	0.912 (0.720, 1.154)	0.441
extended nappers [a]	334	33 (9.9)	0.612 (0.429, 0.873)	**0.007**
**Central obesity**	2272	242 (10.7)		
per-ten minutes increase			0.982 (0.956, 1.008)	0.169
non-nappers [a]	1011	103 (10.2)	1 (ref.)	-
short nappers [a]	227	29 (12.8)	1.218 (0.828, 1.792)	0.317
moderate nappers [a]	699	83 (11.9)	1.085 (0.815, 1.444)	0.577
extended nappers [a]	335	27 (8.1)	0.704 (0.469, 1.055)	0.089

Abbreviations: CI, confidence interval; aRR, adjusted relative risk; MetS, metabolic syndrome; LDL-C, low-density lipoprotein cholesterol; hsCRP, high sensitivity C-reactive protein; HDL-C, high-density lipoprotein cholesterol.

[a] Daytime napping duration of non-nappers:0 minutes/day; Daytime napping duration of short nappers:>0 minutes/day to ≤30 minutes/day; Daytime napping duration of moderate nappers:>30 minutes/day to ≤90 minutes/day; Daytime napping duration of extended nappers:>90minutes/day.

Adjusted for: age, gender, living residence, marital status, educational level, smoking status, drinking status, depressive symptoms, nighttime sleep quality, nighttime sleep duration, serum uric acid, LDL-C , hsCRP and other MetS components.

Table S6. Longitudinal associations of baseline daytime napping duration with the occurrence and remission of MetS after further adjusting for physical activity and multiple drug therapy

Subgroups	Occurrence of MetS, aRR (95% CI)				Remission of MetS, aRR (95% CI)			
	Model 3	*P*	Model 4	*P*	Model 3	*P*	Model 4	*P*
**All subjects**								
per-ten minutes increase	1.014 (1.003, 1.025)	**0.016**	1.014 (1.003, 1.026)	**0.014**	0.991 (0.978, 1.004)	0.188	0.991 (0.978, 1.005)	0.200
non-nappers [a]	Reference	-	Reference	-	Reference	-	Reference	-
short nappers [a]	0.997 (0.835, 1.191)	0.974	0.999 (0.839, 1.189)	0.987	0.995 (0.815, 1.216)	0.964	1.058 (0.871, 1.284)	0.572
moderate nappers [a]	1.087 (0.962, 1.228)	0.182	1.072 (0.949, 1.211)	0.265	0.958 (0.841, 1.091)	0.515	0.984 (0.862, 1.124)	0.815
extended nappers [a]	1.200 (1.036, 1.390)	**0.015**	1.216 (1.050, 1.408)	**0.009**	0.884 (0.727, 1.076)	0.219	0.886 (0.739, 1.063)	0.193
**Sleep duration≥7h**								
per-ten minutes increase	1.023 (1.007, 1.038)	**0.004**	1.022 (1.006, 1.038)	**0.006**	0.981 (0.964, 0.998)	**0.028**	0.981 (0.964, 0.998)	**0.025**
non-nappers [a]	Reference	-	Reference	-	Reference	-	Reference	-
short nappers [a]	0.824 (0.621, 1.093)	0.178	0.834 (0.637, 1.093)	0.189	1.051 (0.808, 1.368)	0.710	1.085 (0.839, 1.403)	0.535
moderate nappers [a]	1.091 (0.921, 1.292)	0.315	1.046 (0.884, 1.238)	0.603	0.968 (0.812, 1.154)	0.717	1.007 (0.840, 1.207)	0.943
extended nappers [a]	1.352 (1.114, 1.641)	**0.002**	1.367 (1.126, 1.660)	**0.002**	0.756 (0.586, 0.976)	**0.032**	0.743 (0.581, 0.952)	**0.019**
**Sleep duration＜7h**								
per-ten minutes increase	1.005 (0.988, 1.021)	0.594	1.006 (0.989, 1.022)	0.497	1.004 (0.984, 1.024)	0.706	1.005 (0.985, 1.026)	0.597
non-nappers [a]	Reference	-	Reference	-	Reference	-	Reference	-
short nappers [a]	1.152 (0.917, 1.447)	0.225	1.145 (0.911, 1.438)	0.245	0.925 (0.697, 1.226)	0.585	0.957 (0.724, 1.266)	0.760
moderate nappers [a]	1.084 (0.907, 1.296)	0.375	1.106 (0.926, 1.321)	0.266	0.940 (0.781, 1.132)	0.516	0.976 (0.804, 1.186)	0.809
extended nappers [a]	1.026 (0.814, 1.294)	0.827	1.034 (0.822, 1.300)	0.777	1.083 (0.832, 1.410)	0.554	1.096 (0.848, 1.417)	0.483
**Good sleep quality**								
per-ten minutes increase	1.020 (1.005, 1.036)	**0.010**	1.019 (1.003, 1.035)	**0.021**	0.985 (0.966, 1.005)	0.144	0.983 (0.966, 1.001)	0.066
non-nappers [a]	Reference	-	Reference	-	Reference	-	Reference	-
short nappers [a]	1.127 (0.879, 1.444)	0.345	1.099 (0.858, 1.408)	0.456	1.002 (0.747, 1.344)	0.990	1.033 (0.779, 1.369)	0.823
moderate nappers [a]	1.072 (0.897, 1.281)	0.447	1.034 (0.866, 1.234)	0.713	0.992 (0.816, 1.206)	0.934	0.999 (0.826, 1.207)	0.989
extended nappers [a]	1.340 (1.101, 1.630)	**0.003**	1.314 (1.078, 1.601)	**0.007**	0.811 (0.614, 1.071)	0.140	0.791 (0.617, 1.013)	0.063
**Poor/Fair sleep quality**								
per-ten minutes increase	1.007 (0.991, 1.023)	0.414	1.009 (0.993, 1.026)	0.260	0.997 (0.977, 1.018)	0.786	1.001 (0.982, 1.020)	0.940
non-nappers [a]	Reference	-	Reference	-	Reference	-	Reference	-
short nappers [a]	0.870 (0.673, 1.125)	0.288	0.898 (0.702, 1.149)	0.390	0.978 (0.742, 1.290)	0.874	1.080 (0.826, 1.411)	0.574

moderate nappers [a]	1.112 (0.940, 1.316)	0.217	1.115 (0.943, 1.318)	0.204	0.911 (0.757, 1.097)	0.324	0.949 (0.787, 1.
extended nappers [a]	1.051 (0.838, 1.319)	0.666	1.102 (0.881, 1.378)	0.396	0.994 (0.734, 1.347)	0.971	1.036 (0.802, 1.

Abbreviations: CI, confidence interval; OR, Odds ratio; MetS, metabolic syndrome; LDL-C, low-density lipoprotein cholesterol; hsCRP, high sensitivity C-reactive protein.

[a] Daytime napping duration of non-nappers:0 minutes/day; Daytime napping duration of short nappers:>0 minutes/day to ≤30 minutes/day; Daytime napping duration of moderate nappers:>30 minutes/day to ≤90 minutes/day; Daytime napping duration of extended nappers:>90minutes/day.

Model 3: Adjusted for physical activity, age, gender, living residence, marital status, educational level, smoking status, drinking status, depressive symptoms, nighttime sleep quality, nighttime sleep duration, serum uric acid, LDL-C and hsCRP.

Model 4: Adjusted for model 3 plus the usage of antihypertensive medicines, lipid-lowering drugs, hypoglycemic agents/insulin and sleeping pills/ anti-depressive treatment.

Table S7. Longitudinal associations of baseline daytime napping duration with the occurrence and remission of MetS according to inverse probability of treatment weighting

Subgroups	Occurrence of MetS		Remission of MetS	
	OR (95% CI)	***P***	OR (95% CI)	***P***
**All subjects**				
per-ten minutes increase	1.017 (1.001, 1.032)	**0.032**	0.989 (0.970, 1.007)	0.227
non-nappers [a]	Reference	-	Reference	-
short nappers [a]	0.957 (0.740, 1.228)	0.735	1.047 (0.791, 1.378)	0.745
moderate nappers [a]	1.055 (0.896, 1.240)	0.520	0.95 (0.785, 1.149)	0.600
extended nappers [a]	1.285 (1.047, 1.573)	**0.016**	0.881 (0.684, 1.128)	0.320
**Sleep duration≥7h**				
per-ten minutes increase	1.032 (1.010, 1.054)	**0.004**	0.971 (0.946, 0.996)	**0.024**
non-nappers [a]	Reference	-	Reference	-
short nappers [a]	0.750 (0.499, 1.101)	0.153	0.990 (0.651, 1.485)	0.962
moderate nappers [a]	1.056 (0.842, 1.322)	0.638	0.982 (0.750, 1.283)	0.894
extended nappers [a]	1.579 (1.200, 2.070)	**0.001**	0.661 (0.463, 0.934)	**0.021**
**Sleep duration＜7h**				
per-ten minutes increase	1.005 (0.982, 1.027)	0.694	0.993 (0.965, 1.021)	0.631
non-nappers [a]	Reference	-	Reference	-
short nappers [a]	1.220 (0.872, 1.689)	0.238	0.870 (0.583, 1.277)	0.484
moderate nappers [a]	1.045 (0.824, 1.321)	0.715	0.851 (0.645, 1.119)	0.251
extended nappers [a]	1.071 (0.784, 1.448)	0.663	0.979 (0.664, 1.426)	0.912
**Good sleep quality**				
per-ten minutes increase	1.025 (1.004, 1.047)	**0.021**	0.979 (0.955, 1.004)	0.102
non-nappers [a]	Reference	-	Reference	-
short nappers [a]	1.069 (0.734, 1.532)	0.722	1.190 (0.788, 1.777)	0.401
moderate nappers [a]	1.015 (0.802, 1.281)	0.903	1.028 (0.782, 1.351)	0.842
extended nappers [a]	1.484 (1.124, 1.952)	**0.005**	0.775 (0.549, 1.085)	0.142
**Poor/Fair sleep quality**				
per-ten minutes increase	1.005 (0.982, 1.027)	0.683	0.991 (0.962, 1.019)	0.525
non-nappers [a]	Reference	-	Reference	-
short nappers [a]	0.886 (0.622, 1.244)	0.495	0.927 (0.624, 1.358)	0.702
moderate nappers [a]	1.061 (0.843, 1.332)	0.610	0.856 (0.652, 1.121)	0.261
extended nappers [a]	1.063 (0.776, 1.441)	0.700	0.937 (0.628, 1.378)	0.744

Abbreviations: CI, confidence interval; OR, Odds ratio; MetS, metabolic syndrome; LDL-C, low-density lipoprotein cholesterol; hsCRP, high sensitivity C-reactive protein.

[a] Daytime napping duration of non-nappers:0 minutes/day; Daytime napping duration of short nappers:>0 minutes/day to ≤30 minutes/day; Daytime napping duration of moderate nappers:>30 minutes/day to ≤90 minutes/day; Daytime napping duration of extended nappers:>90minutes/day.

Adjusted for: age, gender, living residence, marital status, educational level, smoking status, drinking status, depressive symptoms, nighttime sleep quality, nighttime sleep duration, serum uric acid, LDL-C and hsCRP.

Table S8. Longitudinal associations of baseline daytime napping duration with the occurrence and remission of MetS after changing the diagnostic criteria of MetS

Subgroups	Occurrence of MetS		Remission of MetS	
	aRR (95% CI)	*P*	aRR (95% CI)	*P*
**All subjects**				
per-ten minutes increase	1.015 (0.999, 1.031)	0.064	0.989 (0.977, 1.001)	0.066
non-nappers [a]	Reference	-	Reference	-
short nappers [a]	1.030 (0.801, 1.323)	0.821	1.049 (0.880, 1.251)	0.594
moderate nappers [a]	1.080 (0.912, 1.279)	0.374	0.950 (0.839, 1.075)	0.416
extended nappers [a]	1.254 (1.021, 1.540)	**0.031**	0.871 (0.740, 1.026)	0.098
**Sleep duration≥7h**				
per-ten minutes increase	1.018 (0.996, 1.040)	0.104	0.983 (0.969, 0.998)	**0.028**
non-nappers [a]	Reference	-	Reference	-
short nappers [a]	0.768 (0.500, 1.179)	0.227	1.068 (0.850, 1.340)	0.573
moderate nappers [a]	1.010 (0.802, 1.271)	0.933	0.935 (0.797, 1.096)	0.408
extended nappers [a]	1.325 (1.015, 1.729)	**0.039**	0.822 (0.670, 1.009)	0.061
**Sleep duration＜7h**				
per-ten minutes increase	1.013 (0.990, 1.036)	0.277	0.993 (0.976, 1.011)	0.448
non-nappers [a]	Reference	-	Reference	-
short nappers [a]	1.283 (0.939, 1.753)	0.117	1.014 (0.798, 1.290)	0.908
moderate nappers [a]	1.149 (0.899, 1.467)	0.267	0.978 (0.824, 1.161)	0.801
extended nappers [a]	1.204 (0.873, 1.661)	0.257	0.889 (0.699, 1.131)	0.337
**Good sleep quality**				
per-ten minutes increase	1.030 (1.008, 1.051)	**0.007**	0.985 (0.971, 1.000)	**0.048**
non-nappers [a]	Reference	-	Reference	-
short nappers [a]	1.016 (0.700, 1.475)	0.933	1.116 (0.888, 1.404)	0.346
moderate nappers [a]	1.019 (0.794, 1.308)	0.882	0.977 (0.832, 1.147)	0.776
extended nappers [a]	1.608 (1.247, 2.075)	**<0.001**	0.847 (0.691, 1.038)	0.110
**Poor/Fair sleep quality**				
per-ten minutes increase	0.998 (0.976, 1.021)	0.880	0.999 (0.983,1.015)	0.880
non-nappers [a]	Reference	-	Reference	-
short nappers [a]	1.060 (0.760, 1.478)	0.731	1.016 (0.811, 1.273)	0.889
moderate nappers [a]	1.116 (0.890, 1.400)	0.342	1.019 (0.872, 1.191)	0.814
extended nappers [a]	0.932 (0.660, 1.316)	0.688	0.929 (0.739, 1.169)	0.530

Abbreviations: CI, confidence interval; OR, aRR, adjusted relative risk; MetS, metabolic syndrome; LDL-C, low-density lipoprotein cholesterol; hsCRP, high sensitivity C-reactive protein.

[a] Daytime napping duration of non-nappers:0 minutes/day; Daytime napping duration of short nappers:>0 minutes/day to ≤30 minutes/day; Daytime napping duration of moderate nappers:>30 minutes/day to ≤90 minutes/day; Daytime napping duration of extended nappers:>90minutes/day.

Adjusted for: age, gender, living residence, marital status, educational level, smoking status, drinking status, depressive symptoms, nighttime sleep quality, nighttime sleep duration, serum uric acid, LDL-C and hsCRP.

Table S9. Association of daytime napping duration with MetS severity score according to ordinal logistic regression

Subgroups	Subcohort 1 (Individuals Without MetS)		Subcohort 2 (Individuals With MetS)	
	OR (95% CI)	*P*	OR (95% CI)	*P*
**All subjects**				
per-ten minutes increase	1.015 (1.003, 1.026)	**0.013**	1.019 (1.004, 1.035)	**0.012**
non-nappers [a]	Reference	-	Reference	-
short nappers [a]	1.104 (0.923, 1.322)	0.279	1.091(0.871, 1.367)	0.449
moderate nappers [a]	1.091 (0.968, 1.229)	0.152	1.081 (0.927, 1.262)	0.321
extended nappers [a]	1.217 (1.043, 1.420)	**0.012**	1.316 (1.079, 1.606)	**0.007**
**Sleep duration≥7h**				
per-ten minutes increase	1.027 (1.011, 1.044)	**0.001**	1.020 (1.000, 1.041)	**0.046**
non-nappers [a]	Reference	-	Reference	-
short nappers [a]	1.010 (0.778, 1.311)	0.940	1.001 (0.719, 1.393)	0.995
moderate nappers [a]	1.172 (0.993, 1.383)	0.060	0.996 (0.803, 1.235)	0.969
extended nappers [a]	1.423 (1.149, 1.764)	**0.001**	1.368 (1.052, 1.781)	**0.020**
**Sleep duration＜7h**				
per-ten minutes increase	1.001 (0.984, 1.017)	0.942	1.019 (0.995, 1.042)	0.116
non-nappers [a]	Reference	-	Reference	-
short nappers [a]	1.188 (0.926, 1.524)	0.175	1.173 (0.861, 1.597)	0.312
moderate nappers [a]	0.995 (0.837, 1.183)	0.957	1.188 (0.951, 1.484)	0.13
extended nappers [a]	1.025 (0.819, 1.282)	0.829	1.229 (0.903, 1.674)	0.189
**Good sleep quality**				
per-ten minutes increase	1.019 (1.003, 1.035)	**0.018**	1.019 (0.999, 1.040)	0.063
non-nappers [a]	Reference	-	Reference	-
short nappers [a]	1.265 (0.972, 1.645)	0.080	1.050 (0.752, 1.466)	0.776
moderate nappers [a]	1.143 (0.969, 1.348)	0.113	0.993 (0.796, 1.239)	0.953
extended nappers [a]	1.293 (1.050, 1.593)	**0.015**	1.333 (1.024, 1.734)	**0.033**
**Poor/Fair sleep quality**				
per-ten minutes increase	1.010 (0.993, 1.027)	0.239	1.020 (0.997, 1.044)	0.085
non-nappers [a]	Reference	-	Reference	-
short nappers [a]	0.980 (0.766, 1.255)	0.874	1.126 (0.829, 1.529)	0.447
moderate nappers [a]	1.045 (0.879, 1.242)	0.621	1.180 (0.950, 1.465)	0.134
extended nappers [a]	1.157 (0.917, 1.458)	0.218	1.272 (0.928, 1.743)	0.135

Abbreviations: CI, confidence interval; OR, Odds ratio; MetS, metabolic syndrome; LDL-C, low-density lipoprotein cholesterol; hsCRP, high sensitivity C-reactive protein.

[a] Daytime napping duration of non-nappers:0 minutes/day; Daytime napping duration of short nappers:>0 minutes/day to ≤30 minutes/day; Daytime napping duration of moderate nappers:>30 minutes/day to ≤90 minutes/day; Daytime napping duration of extended nappers:>90minutes/day.

Adjusted for: age, gender, living residence, marital status, educational level, smoking status, drinking status, depressive symptoms, nighttime sleep quality, nighttime sleep duration, serum uric acid, LDL-C, hsCRP and MetS severity score at baseline.

Table S10. Longitudinal associations of baseline daytime napping duration with the occurrence and remission of MetS in subgroups by depression, age and gender

Subgroups	Occurrence of MetS		Remission of MetS	
	aRR (95% CI)	*P*	aRR (95% CI)	*P*
**Depressive state**				
per-ten minutes increase	1.011 (0.993, 1.030)	0.235	0.982 (0.959, 1.006)	0.150
non-nappers [a]	Reference	-	Reference	-
short nappers [a]	1.082 (0.801, 1.462)	0.609	0.829 (0.590, 1.166)	0.282
moderate nappers [a]	1.052 (0.853, 1.298)	0.637	0.945 (0.758, 1.179)	0.616
extended nappers [a]	1.201 (0.935, 1.542)	0.152	0.742 (0.517, 1.065)	0.105
**Non-depressive state**				
per-ten minutes increase	1.014 (1.000, 1.028)	0.059	0.995 (0.979, 1.011)	0.548
non-nappers [a]	Reference	-	Reference	-
short nappers [a]	0.982 (0.786, 1.227)	0.875	1.151 (0.905, 1.463)	0.251
moderate nappers [a]	1.089 (0.933, 1.272)	0.281	0.993 (0.836, 1.180)	0.937
extended nappers [a]	1.222 (1.013, 1.473)	**0.036**	0.961 (0.773, 1.195)	0.718
**Age>60**				
per-ten minutes increase	1.019 (1.001, 1.037)	**0.036**	0.982 (0.961, 1.004)	0.109
non-nappers [a]	Reference	-	Reference	-
short nappers [a]	0.870 (0.600, 1.261)	0.462	1.077 (0.791, 1.467)	0.637
moderate nappers [a]	1.249 (1.026, 1.520)	**0.026**	0.911 (0.734, 1.131)	0.399
extended nappers [a]	1.224 (0.956, 1.567)	0.110	0.792 (0.586, 1.070)	0.129
**Age≤60**				
per-ten minutes increase	1.010 (0.995, 1.025)	0.183	0.995 (0.979, 1.012)	0.581
non-nappers [a]	Reference	-	Reference	-
short nappers [a]	1.066 (0.867, 1.310)	0.544	1.040 (0.808, 1.337)	0.762
moderate nappers [a]	0.973 (0.827, 1.146)	0.743	1.004 (0.844, 1.196)	0.961
extended nappers [a]	1.224 (1.015, 1.475)	**0.035**	0.943 (0.749, 1.187)	0.617
**Male**				
per-ten minutes increase	1.019 (1.000, 1.038)	**0.046**	0.992 (0.973, 1.012)	0.448
non-nappers [a]	Reference	-	Reference	-
short nappers [a]	0.995 (0.697, 1.419)	0.976	0.859 (0.606, 1.217)	0.391
moderate nappers [a]	1.222 (0.997, 1.499)	0.054	0.969 (0.790, 1.189)	0.766
extended nappers [a]	1.288 (0.998, 1.661)	0.052	0.896 (0.690, 1.164)	0.412
**Female**				
per-ten minutes increase	1.010 (0.996, 1.025)	0.158	0.990 (0.972, 1.008)	0.260
non-nappers [a]	Reference	-	Reference	-
short nappers [a]	1.018 (0.828, 1.251)	0.869	1.109 (0.877, 1.401)	0.389
moderate nappers [a]	0.964 (0.820, 1.135)	0.661	0.959 (0.801, 1.148)	0.644
extended nappers [a]	1.213 (1.010, 1.458)	**0.039**	0.866 (0.666, 1.127)	0.285

Abbreviations: CI, confidence interval; aRR, adjusted relative risk; MetS, metabolic syndrome; LDL-C, low-density lipoprotein cholesterol; hsCRP, high sensitivity C-reactive protein.

[a] Daytime napping duration of non-nappers:0 minutes/day; Daytime napping duration of short nappers:>0 minutes/day to ≤30 minutes/day; Daytime napping duration of moderate nappers:>30 minutes/day to ≤90 minutes/day; Daytime napping duration of extended nappers:>90minutes/day.

Adjusted for: age, gender, living residence, marital status, educational level, smoking status, drinking status, depressive symptoms, nighttime sleep quality, nighttime sleep duration, serum uric acid, LDL-C and hsCRP.

Table S11. Longitudinal associations of baseline metabolic syndrome status and its components with follow-up daytime napping duration after further adjusting for physical activity and multiple drug therapy

	Model 3		Model 4	
	β (95%CI)	*P* value	*β* (95%CI)	*P* value
**Baseline MetS status**				
Without MetS	Reference		Reference	
With MetS	2.753 (1.367, 4.139)	**<0.001**	2.141 (0.672, 3.610)	**0.004**
**Number of MetS components**				
0 component	Reference		Reference	
1 component	0.238 (-2.165, 2.641)	0.846	0.223 (-2.181, 2.626)	0.856
2 components	2.004 (-0.424, 4.431)	0.106	1.914 (-0.524, 4.353)	0.124
3 components	2.820 (0.247, 5.393)	**0.032**	2.580 (-0.024, 5.184)	0.052
4 components	3.979 (1.089, 6.869)	**0.007**	3.337 (0.333, 6.341)	**0.029**
5 components	7.044 (3.426, 10.662)	**<0.001**	6.034 (2.216, 9.853)	**0.002**
**Baseline MetS components status**				
Without hyperglycaemia	Reference		Reference	
With hyperglycaemia	2.212 (0.879, 3.545)	**0.001**	1.867 (0.497, 3.237)	**0.008**
Without hypertriglyceridemia	Reference		Reference	
With hypertriglyceridemia	2.443 (0.921, 3.966)	**0.002**	1.713 (0.101, 3.326)	**0.037**
Without Low HDL-C	Reference		Reference	
With Low HDL-C	0.286 (-1.054, 1.626)	0.676	-0.104 (-1.462, 1.253)	0.880
Without hypertension	Reference		Reference	
With hypertension	1.957 (0.622, 3.291)	**0.004**	1.587 (0.118, 3.056)	**0.034**
Without central obesity	Reference		Reference	
With central obesity	2.700 (1.299, 4.101)	**<0.001**	2.386 (0.966, 3.806)	**0.001**

Abbreviations: CI, confidence interval; MetS, metabolic syndrome; *β*, regression coefficient; LDL-C, low-density lipoprotein cholesterol; hsCRP, high sensitivity C-reactive protein; HDL-C, high-density lipoprotein cholesterol.
Model 3: Adjusted for physical activity, age, gender, living residence, marital status, educational level, smoking status, drinking status, depressive symptoms, nighttime sleep quality, nighttime sleep duration, serum uric acid, LDL-C, hsCRP and napping duration at baseline in 2011.
Model 4: Adjusted for model 3 plus the usage of antihypertensive medicines, lipid-lowering drugs, hypoglycemic agents/insulin and sleeping pills/ anti-depressive treatment.

Table S12. Longitudinal associations of baseline metabolic syndrome status and its components with follow-up daytime napping duration after using the secondary outcomes

	Nap13-Nap11 [a]		Nap15-Nap11 [b]	
	β (95%CI)	*P* value	*β* (95%CI)	*P* value
**Baseline MetS status**				
Without MetS	Reference		Reference	
With MetS	2.883 (1.086, 4.680)	**0.002**	2.484 (0.740, 4.229)	**0.005**
**Number of MetS components**				
0 component	Reference		Reference	
1 component	0.84 (-2.202, 3.883)	0.588	-0.331 (-3.307, 2.646)	0.828
2 components	3.853 (0.784, 6.921)	**0.014**	0.982 (-2.012, 3.976)	0.520
3 components	3.697 (0.427, 6.968)	**0.027**	2.241 (-0.957, 5.439)	0.170
4 components	6.008 (2.285, 9.732)	**0.002**	2.868 (-0.785, 6.520)	0.124
5 components	7.822 (3.311, 12.332)	**0.001**	4.846 (0.430, 9.263)	**0.032**
**Baseline MetS components status**				
Without hyperglycaemia	Reference		Reference	
With hyperglycaemia	3.118 (1.422, 4.813)	**<0.001**	1.622 (-0.028, 3.272)	0.054
Without hypertriglyceridemia	Reference		Reference	
With hypertriglyceridemia	2.226 (0.261, 4.192)	**0.026**	2.133 (0.230, 4.037)	**0.028**
Without Low HDL-C	Reference		Reference	
With Low HDL-C	0.617 (-1.090, 2.324)	0.479	0.141 (-1.519, 1.801)	0.868
Without hypertension	Reference		Reference	
With hypertension	2.386 (0.683, 4.088)	**0.006**	1.425 (-0.222, 3.071)	0.090
Without central obesity	Reference		Reference	
With central obesity	3.041 (1.232, 4.850)	**0.001**	2.326 (0.571, 4.081)	**0.009**

Abbreviations: CI, confidence interval; MetS, metabolic syndrome; *β*, regression coefficient; LDL-C, low-density lipoprotein cholesterol; hsCRP, high sensitivity C-reactive protein; HDL-C, high-density lipoprotein cholesterol. Adjusted for: age, gender, living residence, marital status, educational level, smoking status, drinking status, depressive symptoms, nighttime sleep quality, nighttime sleep duration, serum uric acid, LDL-C, hsCRP and napping duration at baseline in 2011.

[a]. Differences in napping duration between 2011 and 2013 were used as the secondary outcomes (outcome=nap13-nap11).

[b]. Differences in napping duration between 2011 and 2015 were used as the secondary outcomes (outcome=nap15-nap11).

Table S13. Longitudinal associations of baseline metabolic syndrome status and its components with follow-up daytime napping duration in subgroup analyses

	Subgroups by age		Subgroups by gender	
	>60 years	≤60 years	Male	Female
**Baseline MetS status**				
Without MetS	Reference	Reference	Reference	Reference
With MetS	3.316 (1.063, 5.569)	2.314 (0.548, 4.080)	2.914 (0.680, 5.149)	2.628 (0.855, 4.400)
**Number of MetS components**				
0 component	Reference	Reference	Reference	Reference
1 component	-0.605 (-4.628, 3.419)	0.767 (-2.226, 3.759)	-0.100 (-3.255, 3.054)	0.887 (-2.808, 4.582)
2 components	2.467 (-1.532, 6.466)	1.756 (-1.294, 4.806)	2.326 (-0.959, 5.610)	1.869 (-1.767, 5.505)
3 components	3.469 (-0.743, 7.681)	2.352 (-0.896, 5.599)	3.175 (-0.496, 6.845)	2.778 (-0.960, 6.516)
4 components	3.497 (-1.154, 8.149)	4.515 (0.825, 8.206)	4.211 (-0.150, 8.572)	4.044 (-0.008, 8.095)
5 components	8.980 (3.111, 14.849)	5.384 (0.811, 9.956)	5.706 (-0.202, 11.614)	7.642 (2.832, 12.452)
**Baseline MetS components status**				
Without hyperglycaemia	Reference	Reference	Reference	Reference
With hyperglycaemia	2.884 (0.765, 5.003)	1.698 (-0.026, 3.422)	2.328 (0.337, 4.320)	2.121 (0.325, 3.917)
Without hypertriglyceridemia	Reference	Reference	Reference	Reference
With hypertriglyceridemia	3.256 (0.743, 5.770)	1.840 (-0.078, 3.758)	3.448 (1.109, 5.786)	1.545 (-0.463, 3.552)
Without Low HDL-C	Reference	Reference	Reference	Reference
With Low HDL-C	0.068 (-2.085, 2.220)	0.410 (-1.301, 2.120)	-0.232 (-2.212, 1.747)	0.839 (-0.985, 2.664)
Without hypertension	Reference	Reference	Reference	Reference
With hypertension	2.309 (0.163, 4.454)	1.734 (0.038, 3.429)	2.183 (0.211, 4.154)	1.648 (-0.158, 3.455)
Without central obesity	Reference	Reference	Reference	Reference
With central obesity	3.460 (1.097, 5.824)	2.198 (0.458, 3.938)	1.827 (-0.525, 4.179)	3.258 (1.518, 4.997)

Abbreviations: MetS, metabolic syndrome; LDL-C, low-density lipoprotein cholesterol; hsCRP, high sensitivity C-reactive protein; HDL-C, high-density lipoprotein cholesterol.

Adjusted for: age, gender, living residence, marital status, educational level, smoking status, drinking status, depressive symptoms, nighttime sleep quality, nighttime sleep duration, serum uric acid, LDL-C, hsCRP and napping duration at baseline in 2011.

Table S14: Cross-lagged regression coefficient of daytime napping duration with MetS severity score

Paths	*β*	SE	*P* value	95% CI
Baseline daytime napping duration →Follow-up daytime napping duration	0.382	0.014	<0.001	0.355-0.409
Baseline MetS severity score →Follow-up daytime napping duration	0.037	0.013	0.004	0.011-0.060
Baseline daytime napping duration →Follow-up MetS severity score	0.025	0.011	0.026	0.003-0.047
Baseline MetS severity score →Follow-up MetS severity score	0.583	0.010	<0.001	0.564-0.602
Baseline daytime napping duration ~Baseline MetS severity score	0.092	0.014	<0.001	0.065-0.119
Follow-up daytime napping duration ~Follow-up MetS severity score	0.017	0.010	0.090	-0.003-0.037

→ refers to the lagged effects between variables; ~ refers the covariance between the variables at same time point.

Abbreviation: CI, confidence interval; MetS, metabolic syndrome; *β*, path coefficients (including cross-lagged path coefficients, synchronous correlations and tracking correlations); SE, stand error.

adjusted for: age, gender, living residence, marital status, educational level, smoking status, drinking status, depressive symptoms, nighttime sleep quality, nighttime sleep duration, serum uric acid, LDL-C and hsCRP.
